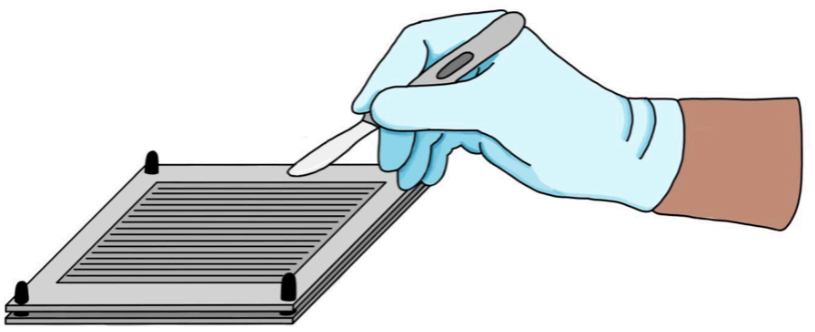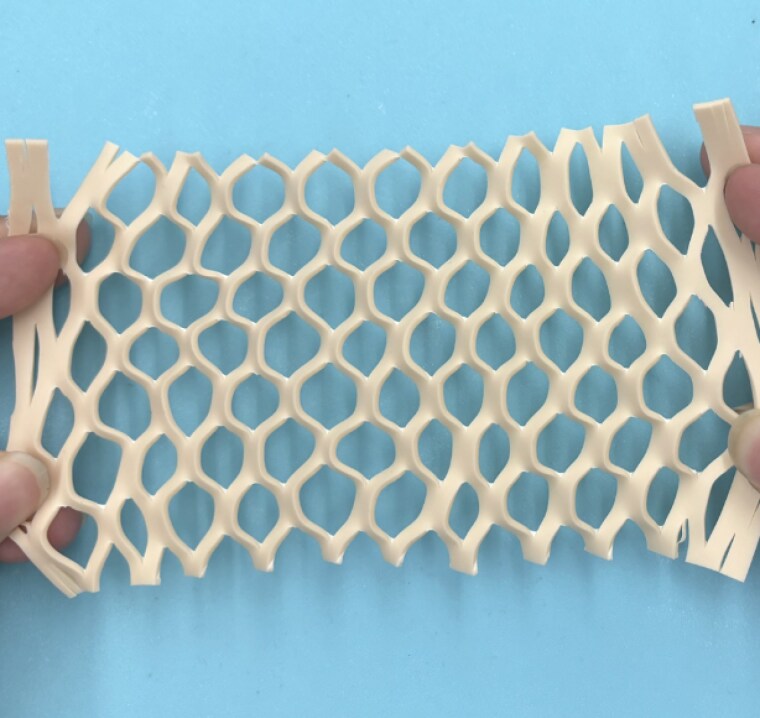# 36 Low-Cost Template for Rapid and Accurate Meshing of Autograft Skin in Surgical Burn Care

**DOI:** 10.1093/jbcr/iraf019.036

**Published:** 2025-04-01

**Authors:** Christine Wang, Ojas Chahal, Aditya Shrinivasan, Aarushi Pant, Ashley Cluff, Dalhart Dobbs, Paree Sharma, Thoya Raman

**Affiliations:** Johns Hopkins Biomedical Engineering, Adult Burn Center; Johns Hopkins University School of Medicine; Johns Hopkins University; Johns Hopkins University; Johns Hopkins University; Johns Hopkins University; Johns Hopkins University; Johns Hopkins University School of Medicine

## Abstract

**Introduction:**

Sub-Saharan Africa (SSA) accounts for 21% of the world’s 180,000 annual burn deaths, despite comprising only 15% of the population. Autologous skin grafting is the standard treatment for severe burns, but for large burns, limited healthy skin can be harvested without risking infection, blood loss, and hypothermia. To cover larger areas, skin must be meshed, but commercial skin meshers are scarce in SSA due to cost, leaving surgeons to hand-mesh with scalpels, which is inefficient for higher expansion ratios. We present a low-cost template for skin meshing, offering a faster, easier alternative in resource-limited settings.

**Methods:**

We created a two-plate template that compresses a skin graft, with the top plate stenciled for parallel scalpel cuts to produce a meshed graft for expansion. Extrusion patterns align the skin for clean, linear cuts. A 3D model was printed using polyethylene terephthalate glycol.

Tattoo practice skin, used to simulate human skin grafts, was meshed by both standard hand-scaling and our template. Time-to-mesh and the consistency of cuts (length and spacing) were measured with ImageJ software. Expansion ratio (final vs. initial length) and graft void area (interstices after meshing) were also quantified.

**Results:**

Results using tattoo practice skin show that the template significantly improves meshing speed and consistency. Meshing a 10cm x 10cm graft with the template took 43 ± 1 seconds, compared to 262 ± 12 seconds by hand. The template achieved an expansion ratio of 1.70:1, higher than the 1.50:1 from hand-meshing, though slightly below the expected 2.13:1. The average void area was 1.33 ± 0.47 cm².

**Conclusions:**

This novel skin graft meshing template offers a more accessible, reusable alternative to commercial meshers for burn care in low-resource settings. It improves speed and achieves expansion ratios up to 1.70:1, addressing critical needs where donor skin is limited. Designed with input from burn surgeons at Johns Hopkins and in Mozambique, it minimizes skin stress and uses standard scalpel blades. Future optimization aims for a 2:1 expansion ratio and reduced void area. This innovation could improve care for 40,000 burn victims annually in sub-Saharan Africa, enhancing treatment outcomes and reducing mortality in underserved regions.

**Applicability of Research to Practice:**

Our low-cost skin graft meshing template has significant real-world applicability, especially in resource-limited regions like sub-Saharan Africa. It reduces meshing time (from over 4 minutes to under 1 minute), increases expansion ratios (up to 1.70:1), and uses accessible materials, making it easy for surgeons to adopt with minimal training. This research directly translates into improved burn care, potentially reducing morbidity and mortality in underserved areas. It also serves as a model for future medical innovations designed for low-resource settings, improving patient outcomes and care standards.

**Funding for the Study:**

N/A